# Inferring lumbar lordosis in Neandertals and other hominins

**DOI:** 10.1093/pnasnexus/pgab005

**Published:** 2022-03-02

**Authors:** Scott A Williams, Iris Zeng, Glen J Paton, Christopher Yelverton, ChristiAna Dunham, Kelly R Ostrofsky, Saul Shukman, Monica V Avilez, Jennifer Eyre, Tisa Loewen, Thomas C Prang, Marc R Meyer

**Affiliations:** Center for the Study of Human Origins, Department of Anthropology, New York University, New York, NY 10003, USA; New York Consortium in Evolutionary Primatology, New York, NY 10024, USA; Centre for the Exploration of the Deep Human Journey, University of the Witwatersrand, Private Bag 3, Wits 2050, Johannesburg, South Africa; Evolutionary Studies Institute, University of the Witwatersrand, Private Bag 3, Wits 2050, Johannesburg, South Africa; Department of Architecture, Massachusetts Institute of Technology, Cambridge, MA 02139, USA; Department of Chiropractic, Faculty of Health Sciences, University of Johannesburg, 2094, Johannesburg, South Africa; Evolutionary Studies Institute, University of the Witwatersrand, Private Bag 3, Wits 2050, Johannesburg, South Africa; Department of Chiropractic, Faculty of Health Sciences, University of Johannesburg, 2094, Johannesburg, South Africa; Center for the Study of Human Origins, Department of Anthropology, New York University, New York, NY 10003, USA; Department of Anthropology, Texas State University, San Marcos, TX 78666, USA; Department of Anatomy, College of Osteopathic Medicine, New York Institute of Technology, Old Westbury, NY 11569, USA; Center for the Study of Human Origins, Department of Anthropology, New York University, New York, NY 10003, USA; Center for the Study of Human Origins, Department of Anthropology, New York University, New York, NY 10003, USA; New York Consortium in Evolutionary Primatology, New York, NY 10024, USA; Center for the Study of Human Origins, Department of Anthropology, New York University, New York, NY 10003, USA; Department of Anthropology, Bryn Mawr College, Bryn Mawr, PA 19010, USA; School of Human Evolution and Social Change, Arizona State University, Tempe, AZ 85287, USA; Department of Anthropology, Texas A&M University, College Station, TX 77843, USA; Department of Anthropology, Chaffey College, Rancho Cucamonga, CA 91737, USA

## Abstract

Lumbar lordosis is a key adaptation to bipedal locomotion in the human lineage. Dorsoventral spinal curvatures enable the body's center of mass to be positioned above the hip, knee, and ankle joints, and minimize the muscular effort required for postural control and locomotion. Previous studies have suggested that Neandertals had less lordotic (ventrally convex) lumbar columns than modern humans, which contributed to historical perceptions of postural and locomotor differences between the two groups. Quantifying lower back curvature in extinct hominins is entirely reliant upon bony correlates of overall lordosis, since the latter is significantly influenced by soft tissue structures (e.g. intervertebral discs). Here, we investigate sexual dimorphism, ancestry, and lifestyle effects on lumbar vertebral body wedging and inferior articular facet angulation, two features previously shown to be significantly correlated with overall lordosis in living individuals, in a large sample of modern humans and Neandertals. Our results demonstrate significant differences between postindustrial cadaveric remains and archaeological samples of people that lived preindustrial lifestyles. We suggest these differences are related to activity and other aspects of lifestyle rather than innate population (ancestry) differences. Neandertal bony correlates of lumbar lordosis are significantly different from all human samples except preindustrial males. Therefore, although Neandertals demonstrate more bony kyphotic wedging than most modern humans, we cast doubt on proposed locomotor and postural differences between the two lineages based on inferred lumbar lordosis (or lack thereof), and we recommend future research compare fossils to modern humans from varied populations and not just recent, postindustrial samples.

Significance Statement Lumbar lordosis is a primary adaptation to bipedal locomotion in hominins. Based on their skeletal remains, Neandertals have long been thought to lack modern human-like lordosis, instead possessing relatively straight lower backs lacking significant ventral curvature (i.e. the “small of the back”). However, the modern human samples that Neandertals have been compared to are largely recent postindustrial specimens. These differ significantly from Neandertals, whereas sex-specific preindustrial lifestyle samples of modern humans do not. Given that lumbar lordosis is formed in part by soft tissue structures (e.g., intervertebral discs) that respond to activity and affect bony contributions to lumbar lordosis, Neandertals and other fossil hominins are best compared to preindustrial (i.e. less sedentary and more active) modern human samples.

## Introduction

Modern humans (*Homo sapiens*) demonstrate lumbar lordosis, a ventral convexity of the lower back that counters the kyphotic curve (ventral concavity of the upper back) and contributes to the sinusoidal curvature of the human spine. This configuration evolved to balance and stabilize the upright trunk over two legs and dissipate loads through the vertebral column, pelvis, and lower limbs during bipedal posture and locomotion. Curvatures of the spine are produced not only by the wedging of vertebrae and angulation of the articular processes and of the sacrum within the pelvis (pelvic incidence) (1, 2), but also by soft tissue structures such as intervertebral discs, which can change in shape over the course of a single day (3). As intervertebral discs and intervertebral joints are flexible, the position in which the body is held can also affect overall measurements of spinal curvature (4). Clinical studies on modern human spinal curvature are abundant, and several techniques for quantifying lordosis on lateral radiographs have been formalized (reviewed in ref. (4)). However, the fragmentary nature of the fossil record does not allow the direct application of these methods; therefore, researchers have relied on the wedging of lumbar vertebrae and other osteological correlates of lordosis to extrapolate the degree of lumbar lordosis in fossil hominins (1, 5–10).

In humans, sexual dimorphism in the wedging of lumbar vertebrae has been linked to the capacity for pregnancy in women (11–13). The overall lordosis angle (“Cobb angle,” LA), measured on radiographs of living individuals, differs in standing and supine postures, where the lumbar spine is extended and flattened, respectively (4, 13). Therefore, flexible soft tissues and their effect on posture play a major role overall lordosis. In fact, intervertebral discs can change significantly over the course of just an hour of running (3). Vertebral body wedging is less flexible, particularly in the short term, and is unaffected by an individual's posture. In contrast, varying measurements of LA have resulted in ambiguity in the literature on the existence of sexual dimorphism in lumbar lordosis (4, 13). Studies based on vertebral body wedging are less ambiguous in demonstrating that females are characterized by significantly more dorsal (lordotic) wedging than males on average (9, 11, 13–15).

Previous research on variation across recent modern humans suggests that populations differ substantially in degree of vertebral body wedging (9, 14, 16, 17). By and large, these studies have suggested that Europeans, Middle Easterners, and European Americans tend to demonstrate more dorsal wedging of lumbar vertebrae than Africans, island Southeast Asians, Australasians, and Native Americans. However, in these studies, postindustrial cadaveric (i.e. from medical schools and other 19th and 20th century skeletal collections) European, Middle Eastern, and European American samples are often compared with noncadaveric, archaeological samples of hunter–gatherers from other regions. In studies comparing different groups of postindustrial remains (e.g. African Americans and European Americans), significant differences were not found (17, 18). Recently, García-Martínez et al. (9) cautioned that intercontinental (i.e. Africa–Asia–Europe) variation is greater than within-population sex differences, but their continental groups conflated mixed lifestyle samples (e.g. postindustrial cadaveric vs. hunter–gatherer archaeological remains). Given different lifestyles (subsistence practices, activity levels, reliance of furniture, and so on) and vertebral loading regimes, differences in bony correlates of lumbar lordosis may be expected.

Outside of *H. sapiens*, other hominin species are also thought to have been characterized by lumbar lordosis, inferred necessarily from fossilized remains (5, 10, 11, 19, 20). Interestingly, the presence of lumbar lordosis in one of our closest relatives, Neandertals, is debated. Boule (21) described the spinal curvature of the first-discovered Neandertal vertebral column (La Chapelle-aux-Saints 1) as ape-like in many ways, including a smaller degree of lumbar lordosis than commonly observed in modern humans. Boule's reconstruction was both supported and criticized (22, 23), and now, multiple Neandertal skeletons with partial vertebral columns are known, including Kebara 2 and Shanidar 3, which include the most complete lumbar columns (24, 25). Weber and Pusch (7) (p. S329) reported an overall assessment of “a natural lumbar kyphosis” in these two Neandertal lumbar columns, contrasting them with the normal, nonpathological condition in modern humans. In Kebara 2 and Shanidar 3, only the last lumbar vertebra is dorsally wedged, as is also the case with La Chapelle-aux-Saints 1 (21). Indeed, most studies infer Neandertal lumbar lordosis as much less pronounced than in modern humans or absent entirely (1, 5–8, 21, 26). Been et al. (5, 26) have also inferred postural, weight-bearing, and locomotor differences between the Neandertal and modern human lineages based on differences in lumbar wedging.

The most appropriate comparisons to Neandertals (and other fossil hominins) would be penecontemporaneous (Middle–Late Pleistocene) modern humans; however, preserved vertebral columns of this group are relatively few in number. McCown and Keith (27) describe the early Late Pleistocene Skhūl material, which includes the bodies of L2-5 of one individual (Skhūl IV) and L4-5 of another (Skhūl VII). As with the Neandertals, only the last lumbar vertebra is dorsally wedged (27) (p. 106): “Boule inferred that the lumbar region in the European Neanderthals was straight; the evidence at our disposal points to the same condition in the Skhūl people.” Been et al. (5) report modern human-like dorsal wedging of multiple elements in a combination of Crô-Magnon *H. sapiens* material less than half the age of the Skhūl people, but the association of these vertebrae (from Crô-Magnon 1, 2, and 3) is a new proposal that contrasts with previous reports (28, 29) and is questionable on morphological grounds (SAW, personal observation).

Most studies have relied on 20th century European, Middle Eastern, and nonindigenous U.S. American cadaveric (hereafter, “postindustrial”) samples in comparative studies of Neandertals and other fossil hominins (1, 5, 7, 8, 26), but see (6, 9).The goal of this study is not to quantify the overall curvature of the lower back in living or extinct hominins, which would require the inclusion of soft tissue (nonexistent in fossil hominins), but rather to compare bony contributions of lordosis among fossil hominins with a more varied set of recent modern human populations. We test for differences among human groups, defined by sex, geography/ancestry, and context (postindustrial lifestyles vs. hunter–gatherer lifestyles; hereafter, “preindustrial”), and we compare Neandertals to modern human groups to test the null hypothesis that there are no differences among Late Pleistocene hominins in the bony wedging of lumbar vertebrae. Differences in bony aspects of lumbar lordosis between modern human lifestyle types would indicate that plasticity plays a significant role in lumbar vertebra morphology. In contrast, differences in geography/ancestry irrespective of lifestyle might indicate that local adaptation (i.e. via natural selection) explains variation in lumbar lordosis among human populations as suggested by previous work.

## Materials and Methods

**Fig. 1.**
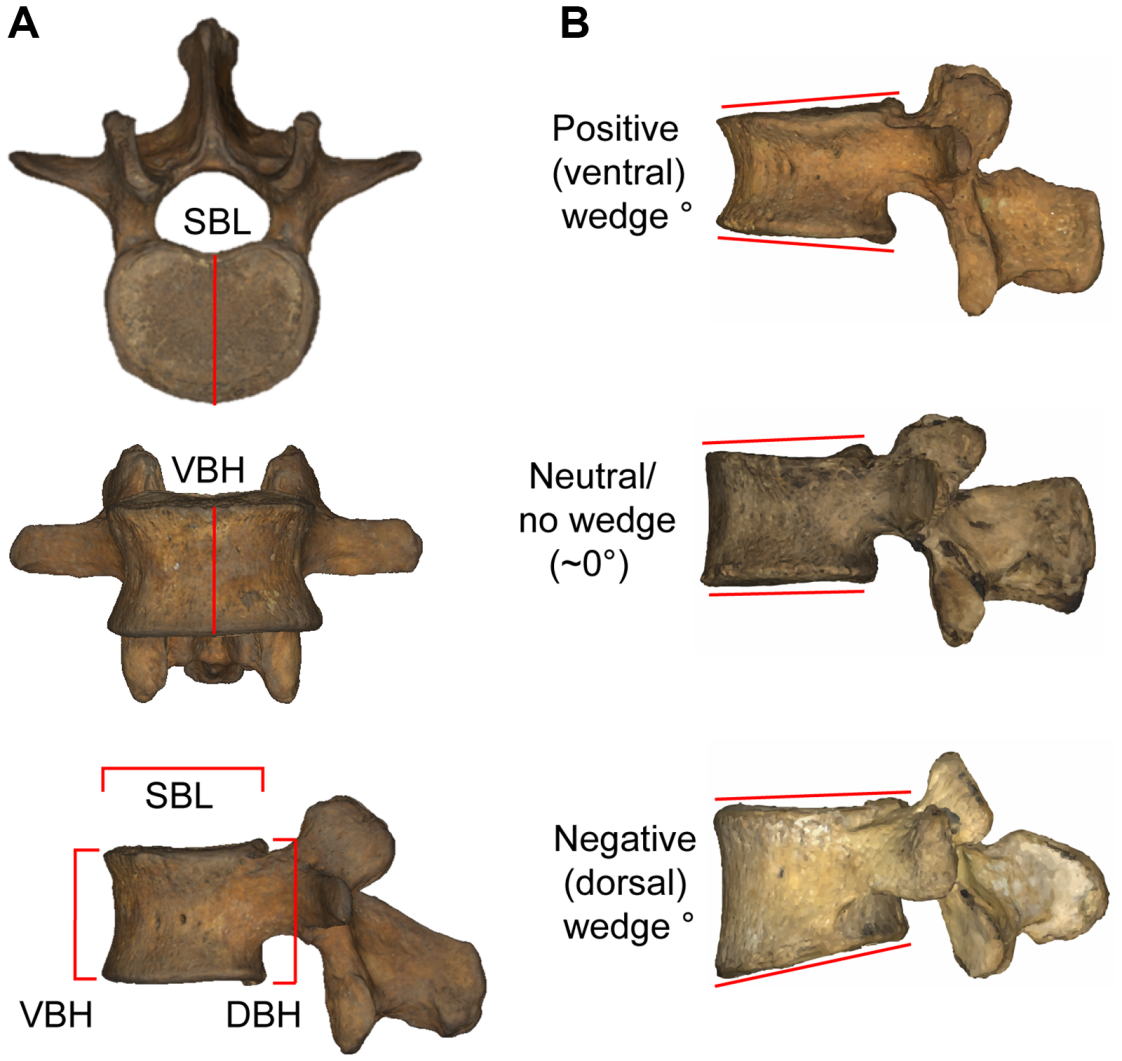
Measurements taken on lumbar vertebrae and three types of WAs. (**A**) Depictions of the three measurements collected for this study: superior vertebral body dorsoventral length (SBL), ventral vertebral body superior–inferior height (VBH), dorsal vertebral body superior–inferior height (DBH). (**B**) Ventral (kyphotic) wedging (positive WAs), neutral wedging (WA ∼0°), and dorsal (lordotic) wedging (negative WAs).

Data were collected on human skeletal material at the following institutions: Dart collection, University of the Witwatersrand; American Museum of Natural History; Cleveland Museum of Natural History; Florisbad Quarternary Research Station, National Museum, Bloemfontein; Natural History Museum; Musée de l'Homme; University of Tennessee Knoxville (UTK); and Texas State University (TS). Mitutoyo digital calipers (Mitutoyo Inc., Japan) were placed along the superior and inferior aspects of the vertebral body along the midline at its ventral- and dorsal-most extensions. A third measurement was taken with calipers placed on the ventral-most aspect of the spinal canal at the midline and at the ventral-most aspect of the superior vertebral body at midline. The resulting three measurements (ventral vertebral body superior–inferior height: VBH, dorsal vertebral body superior–inferior height: DBH, and superior vertebral body dorsoventral length: SBL; Figure 1) yield the WA: 2*arctangent (((DBH–VBH)/2)/SBL). We report WA at individual lumbar levels and also calculate a sum L1-L5 WA value (∑WA) for each specimen (6). Meyer (20) introduced the inferior articular process lateral angle (AP), which he linked to lumbar lordosis. Been et al. (1) used a slightly modified version of AP (measured along the anterior extent of the inferior articular facet rather than through its long axis as in ref. (21)) and found their sum (∑AP) to correlate more strongly with LA than ∑WA, although both ∑WA and ∑AP are significantly correlated with LA. For consistency with the most recent literature, we follow Been et al.’s (1) method of quantifying AP (Figure 1).

All five lumbar vertebrae were measured for WA, amounting to 1,660 vertebrae represented by 332 modern human individuals (*N*_Females_ = 158; *N*_Males_ = 174). Only adult individuals were measured for this study. Postindustrial specimens with recorded ages below 18 years were not included, nor were preindustrial specimens without fused epiphyseal rings on the vertebrae. We also excluded individuals with recorded age above 55 years and those individuals presenting spondyloses or other degenerative pathologies affecting the dimensions of the vertebral body. Specimens without recorded sex were categorized as male or female using standard sexing techniques on associated skeletal material (30). A subsample of specimens (from the preindustrial sample and two postindustrial collections: UTK and TS) preserving intact inferior articular processes were measured for AP, including some additional individuals that exceeded the age of 55 years to maximize sample sizes. We test for age differences to confirm that AP is not influenced by old age (< 56 years of age vs. > 55 years of age).

Our hypothesis testing is necessarily hierarchical. Sexual dimorphism in lumbar vertebral wedging has long been demonstrated (9, 11, 14, 15, 31), so we group by sex and test the null hypothesis that sexes are not significantly different in WA. We also categorize samples as preindustrial (*N*_Total_ = 78: *N*_F_ = 31; *N*_M_ = 47) and postindustrial (*N*_T_ = 254: *N*_F_ = 127; *N*_M_ = 127) and test the null hypothesis that lifestyle differences are not associated with significant differences in WA. Our postindustrial sample is split into African ancestry (Black South Africans: *N*_T_ = 102: *N*_F_ = 60; *N*_M_ = 42; African Americans: *N*_T_ = 29: *N*_F_ = 19; *N*_M_ = 10) and European ancestry (White South Africans: *N*_T_ = 43: *N*_F_ = 17; *N*_M_ = 26; European Americans: *N*_T_ = 78: *N*_F_ = 30; *N*_M_ = 48). Due to sample size restrictions, we split our preindustrial data into the following groups to test for geographical differences: African ancestry (*N*_T_ = 39: *N*_F_ = 16; *N*_M_ = 23) and Asia–Pacific/South American ancestry (*N*_T_ = 39: *N*_F_ = 15; *N*_M_ = 24). We use these geographical ancestry categories to test the null hypothesis that postindustrial samples do not differ significantly in WA. For the AP analysis, our postindustrial samples are only derived from UTK and TS and are restricted to recent Americans of European ancestry (*N*_T_ = 89: *N*_F_ = 36; *N*_M_ = 53); our preindustrial lifestyle sample is limited to smaller sample sizes of African (*N*_T_ = 27: *N*_F_ = 9; *N*_M_ = 18) and Asia–Pacific/South American ancestries (*N*_T_ = 34: *N*_F_ = 13; *N*_M_ = 21). Therefore, we test the same hypotheses outlined above for the AP data, but do not test for differences in geographic ancestry of the postindustrial sample since our dataset contains only one group (European Americans).

Very few fossil hominins preserve a lumbar column complete enough to calculate ∑WA and ∑AP. Two male Neandertals preserve complete lumbar bodies (Kebara 2 and Shanidar 3) and partial lumbar columns of fossil modern humans (Crô-Magnon and Skhūl) are included in plots of individual lumbar levels (*N* = 25 fossil vertebrae in total). For ∑AP estimates, only Shanidar 3 and Kebara 2 preserve the necessary morphology. Both Neandertals are inferred to be males; therefore, they are pooled here for comparisons with modern human samples. The Shanidar 3 Neandertal has just four lumbar vertebrae when the absence of costal facets is used as the criterion; the fifth to last presacral vertebra bears costal facets but sagittalized, lumbar-like zygapophyses (Figure S3, Supplementary Material), so we include it here as L1. We also analyze individual fossil lumbar vertebrae and partial fossil lumbar columns for WA, including La Chapelle-aux-Saints 1 (male Neandertal), Crô-Magnon 1 and 3 (2+ individuals possibly of mixed sex; see (5) for a different association), and Skhūl IV and VII (inferred male and female fossil modern humans (27)).

We test for normality using Shapiro-Wilk tests and use ANOVA with Tukey's pairwise comparisons to test the null hypothesis that groups are not different from each other if the data are normal; if they are non-normal, we use nonparametric ANOVA (Kruskal–Wallis). One-way ANOVA is used on each factor (sex, lifestyle, and geography/ancestry) separately. Additionally, two-way ANOVA is carried out on the factors sex and lifestyle together and sex and geography/ancestry together. A standard alpha level of 0.05 is used to represent statistical significance. We report both 95% CIs of the mean, estimated using a Bootstrap resampling procedure with 9999 replicates, and 95% PIs of the mean (1.96 × SD).

## Results

All data are normally distributed (*P* > 0.05); consequentially, we use parametric tests throughout our analyses. To summarize our methods, one-way ANOVA tests with Tukey's pairwise comparisons were run to test three null hypotheses for both vertebral body wedging angle (WA) and inferior articular process angle (AP) datasets that the following groups are not significantly different from one another: sexes, geographic ancestries, and context of the remains (preindustrial vs. postindustrial). We also tested a null hypothesis that differences in young adult/middle age (18-55 years of age) and old age (> 55 years of age) groups for AP data were not significantly different from each other to potentially pool the two age groups for subsequent analyses. For WA analyses, older-aged (> 55 years) postindustrial specimens were not included to avoid degenerative pathologies, nor were individuals with significant pathologies of the vertebral bodies in the preindustrial sample. Additionally, two-way ANOVA tests with Tukey's pairwise comparisons were conducted on the sum of lumbar vertebral body WAs (∑WA) and the sum of lumbar inferior APs (∑AP), with sex and lifestyle (preindustrial and postindustrial) as factors.

The two-way ANOVA tests on ∑WA and ∑AP demonstrate that both sex and lifestyle are significant factors (*P* ≤ 0.001), whereas geography/ancestry is not (*P* > 0.05). The remainder of the results presented below are generated from one-way ANOVA tests.

### Sex differences in WA and AP

**Fig. 2.**
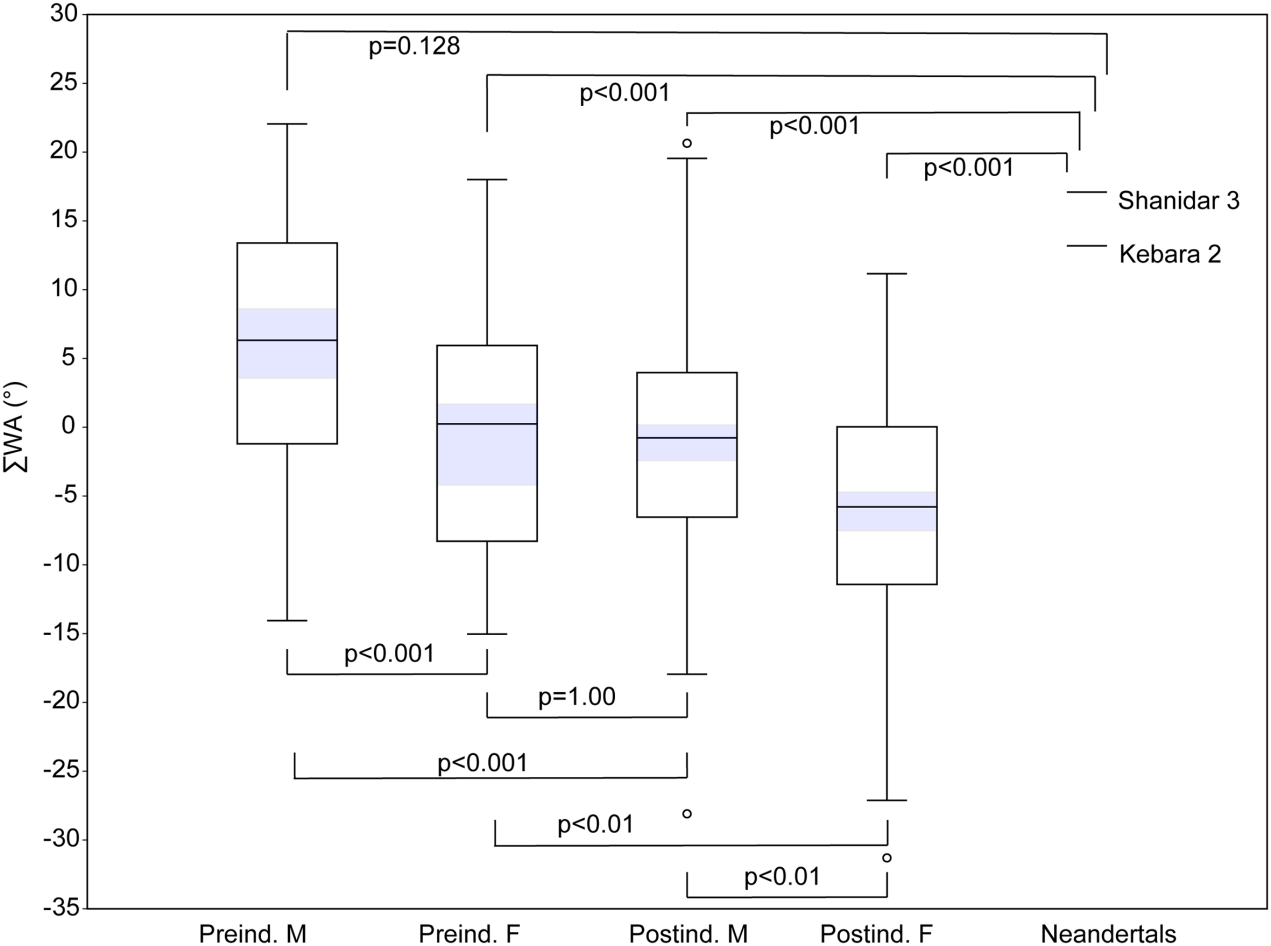
Sum of lumbar vertebral body WAs (∑WA) in modern humans and fossil hominins. The sex-specific postindustrial and preindustrial samples differ significantly, with postindustrial samples demonstrating more dorsal (lordotic) wedging than preindustrial samples. The female preindustrial and male postindustrial samples do not differ significantly. The small male Neandertal sample differs significantly from all but the male preindustrial sample. The shaded areas represent the 95% CIs of the means.

**Fig. 3.**
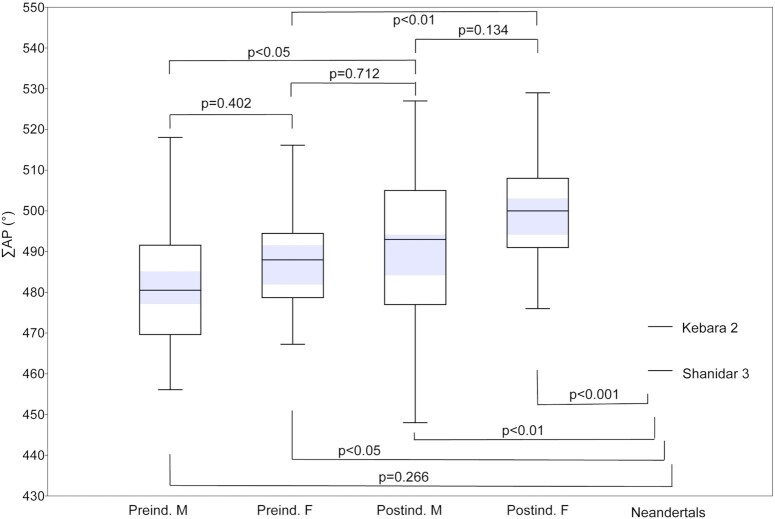
Sum of lumbar inferior APs (∑AP) in modern humans and fossil hominins. The sex-specific preindustrial and postindustrial samples differ significantly, with postindustrial samples demonstrating higher angles (more dorsal projection of the inferior articular processes) than preindustrial samples. Sexes within samples do not differ significantly, nor do female preindustrial and male postindustrial samples. The small male Neandertal sample differs significantly from all but the male preindustrial sample. The shaded areas represent the 95% CIs of the means.

Our postindustrial sample demonstrates sex differences in the sum of lumbar vertebral body WAs (∑WA) and at all 5 individual lumbar vertebral levels (*P* < 0.05; Figure 2; Figure S1, Supplementary Material). In our less well-sampled preindustrial sample, only ∑WA (Figure 2) and WA at individual levels L1 and L2 demonstrate significant sex differences (*P* < 0.05; Figure S1 and Tables S1–S5, Supplementary Material). WA at levels L3 (*P* = 0.093), L4 (*P* = 0.134), and L5 (*P* = 0.378) are not significantly different, although the same pattern as in the postindustrial sample can be observed in the preindustrial sample (Figure S1, Supplementary Material). For the sum of lumbar inferior APs (∑AP), sexes are not significantly different (*P* > 0.05) except the male preindustrial and female postindustrial samples (*P* < 0.001; Figure 3).

### Lifestyle differences in WA and AP

The preindustrial and postindustrial lifestyle samples show significant differences in ∑WA (*P* < 0.05) and across individual levels excluding the last lumbar (L5), with one exception: the female L1 samples are not significantly different from each other (*P* = 0.263; Figure 2; Figure S1 and Tables S1–S5, Supplementary Material). Otherwise, male L1 and male and female L2, L3, and L4 WA levels are significantly different (*P* < 0.05), with the postindustrial samples demonstrating lower (more dorsal) wedging values. At the L5 level, preindustrial and postindustrial samples are not significantly different in either males (*P* = 0.842) or females (*P* = 0.564). The female preindustrial sample is not significantly different from the male postindustrial sample at any level or in ∑WA (*P* > 0.05). For ∑AP, in both sexes, the postindustrial group produces significantly higher angle measurements (more dorsal deflection of the inferior articular process) than the preindustrial lifestyle group (females *P* = 0.007; males *P* = 0.043; Figure 3).

### Geographic ancestry and age differences in WA and AP

We analyze ∑WA and find no differences in the male (*P* = 0.994) or female (*P* = 0.294) African and Asia Pacific/South American preindustrial samples (Figure S2A, Supplementary Material). Among postindustrial samples, we find no significant differences between African ancestry (N_T_ = 131: N_F_ = 79, N_M_ = 52) and European ancestry (N_T_ = 119: N_F_ = 47, N_M_ = 72) groups in either males (*P* = 0.228) or females (*P* = 0.328; Figure S2B, Supplementary Material). Likewise, for ∑AP, we find no differences in geographic ancestry for the preindustrial sample in either males (*P* = 0.939) or females (*P* = 0.818; Figure S2C, Supplementary Material). In the postindustrial sample, we are unable to test for geographic ancestry differences, but we do test for differences in ∑AP due to age. We do not find significant differences in our age categories (< 56 vs. > 55) for either males (*P* = 0.431) or females (*P* = 0.940; Figure S2D, Supplementary Material). Therefore, pooling of age groups for postindustrial ∑AP and geographic ancestry for the preindustrial ∑AP and WA is appropriate.

### Neandertals

**Fig. 4.**
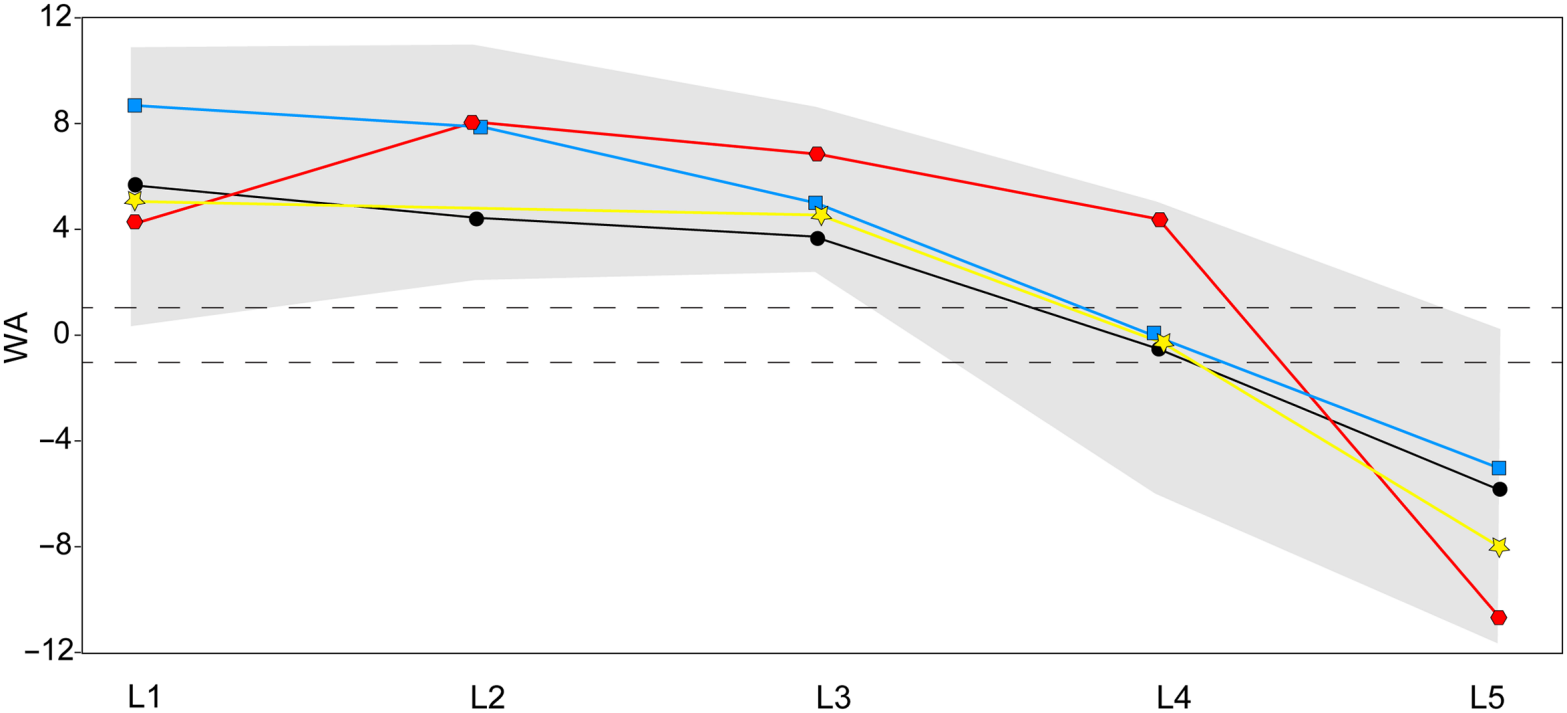
Neandertal vertebral body WA plotted with the male preindustrial modern human sample (black circles with 95% PIs of the means shown with shading). Kebara 2 (red hexagons), Shanidar 3 (blue squares), and La Chapelle-aux-Saints 1 (yellow stars) are not distinct in magnitude or pattern of WAs than male preindustrial modern humans. The dashed lines outline a zone of relatively neutral wedging (−1° to 1°). La Chapelle-aux-Saints 1 does not preserve a second lumbar vertebra; therefore, a line is used to connect the L1 and L3 data points.

Neandertal ∑WA and ∑AP fall outside of the female postindustrial sample but within the range of all other human groups (Figures 2 and 3). Neandertals are significantly different (*P* < 0.05) from all human groups except the male preindustrial sample for both ∑WA (*P* = 0.128) and ∑AP (*P* = 0.266). At individual WA levels, Neandertals fall within the range of variation of the four human groups, with the following exceptions: Shanidar 3 is outside the range of variation of human females at L1, Kebara 2 and Shanidar 3 are outside the range of all but the male preindustrial sample at L2, Shanidar 3 falls outside the range of the postindustrial human samples at L3, Kebara 2 falls outside all but the male preindustrial sample at L3, and Kebara 2 falls outside the range of the postindustrial human samples at L4 (Figure S1, Supplementary Material). In all the aforementioned cases, Neandertals demonstrate high (kyphotic) angles. Neandertal ∑WA and ∑AP are situated on the high end of human variation but fit comfortably within the range and 95% PIs of preindustrial modern human males (Tables 1 and  2; Figures 2  and 3). At individual lumbar levels, the Neandertal specimens fall within the 95% PIs of the male preindustrial sample and conform to the pattern of change across levels observed in this sample (Figure 4).

**Table 1.** Descriptive statistics of ∑WA.

Group	*N*	Mean (SD)	95% PI lower	95% PI upper
Postindustrial	254			
Females	127	−6.13 (8.16)	−22.12	9.86
Males	127	−1.13 (7.46)	−15.75	13.49
Preindustrial	78			
Females	31	−1.21 (8.66)	−18.17	15.75
Males	47	6.07 (9.06)	−11.69	23.83
Neandertals	2	15.15 (2.76)	9.74	20.56

**Table 2.** Descriptive statistics of ∑AP.

Group	*N*	Mean (SD)	95% PI lower	95% PI upper
Postindustrial	89			
Females	36	498.61 (13.45)	472.25	524.97
Males	53	489.19 (18.26)	453.41	524.98
Preindustrial	61			
Females	22	486.86 (11.55)	464.23	509.49
Males	40	481.18 (13.04)	455.62	506.74
Neandertals	2	466.19 (7.65)	451.20	481.81

## Discussion

Our sample is divided into postindustrial (20th century cadaveric) and preindustrial lifestyle (archaeological hunter–gatherer) human remains. We find that both sex and lifestyle are associated with differences in vertebral body WAs at most lumbar vertebral levels and in the sum of lumbar vertebral body WAs (∑WA) and the sum of lumbar inferior APs (∑AP), with females of both lifestyles and postindustrial samples of both sexes demonstrating more dorsal wedging than males and the preindustrial samples (Figures 2 and 3). Importantly, the female preindustrial sample is not significantly different from the male postindustrial sample for ∑AP, ∑WA, and WA at individual levels, suggesting that preindustrial and postindustrial material should be treated separately (Figures 2 and 3; Figure S1, Supplementary Material). Additionally, we find that although Neandertals demonstrate high WA, especially at upper lumbar levels, along with atypical ∑WA and ∑AP for modern humans, they are not outside the modern human range of variation and in fact are not significantly different from the male preindustrial sample for either ∑WA or ∑AP (Tables 1 and 2).

Cunningham (14) and others (9, 16, 17, 31) suggested that geographic variation in lumbar wedging patterns exist across human populations. Turner (16) (p. 542) took a very typological approach to human variation and identified three “types” of lower backs among humans: lordotic (“*Kurto-rachic*,” sum dorsal wedging), kyphotic (“*Koilo-rachic*,” sum ventral wedging), and straight spines (“*Ortho-rachic*;” relatively neutral wedging), which he argued varyingly characterized different “races.” Our results demonstrate that “spine types” corresponding to “racial types” do not exist; rather, all categories (sex, temporal, and geographic) we examined contain individuals that would be classified into all three spine types (Figure 2). The mean sum of WAs for the female preindustrial and male postindustrial samples fall close to −1°, which can be seen as a “neutral wedging” (i.e. “straight spine”) zone, whereas the mean of our male preindustrial sample (6°) represents ventral wedging and the mean of our female postindustrial sample (−6°) represents dorsal wedging. However, continuous variation exists, with extensive overlap between all four groups (Figure 2). Similar patterns are seen at individual lumbar levels (Figure S1, Supplementary Material) and between sexes (where female average WA are almost always lower than those of males) throughout (Figure S1, Supplementary Material).

Typological interpretations of 19th century researchers presumed that bony wedging of lumbar vertebrae was an evolved feature of geographic races due to adaptations of the spine to flexibility and stability. Cunningham (14) (p. 35) argued that Europeans “sacrificed in the lumbar part of the vertebral column flexibility for stability,” whereas “the savage… who is frequently called upon to pursue game in a supine position, and climb trees in search of fruit, preserves the pithecoid condition of vertebra in lumbar region; and on account of this a superior flexibility of the spine must result.” Cunningham (14) rejected the influence of plastic change of vertebral body shape during one's lifetime, stating “it cannot be due to an immediate and mechanical influence operating upon the vertebral bodies during the life of the individual…. It is an hereditary condition” (p. 379).

**Fig. 5.**
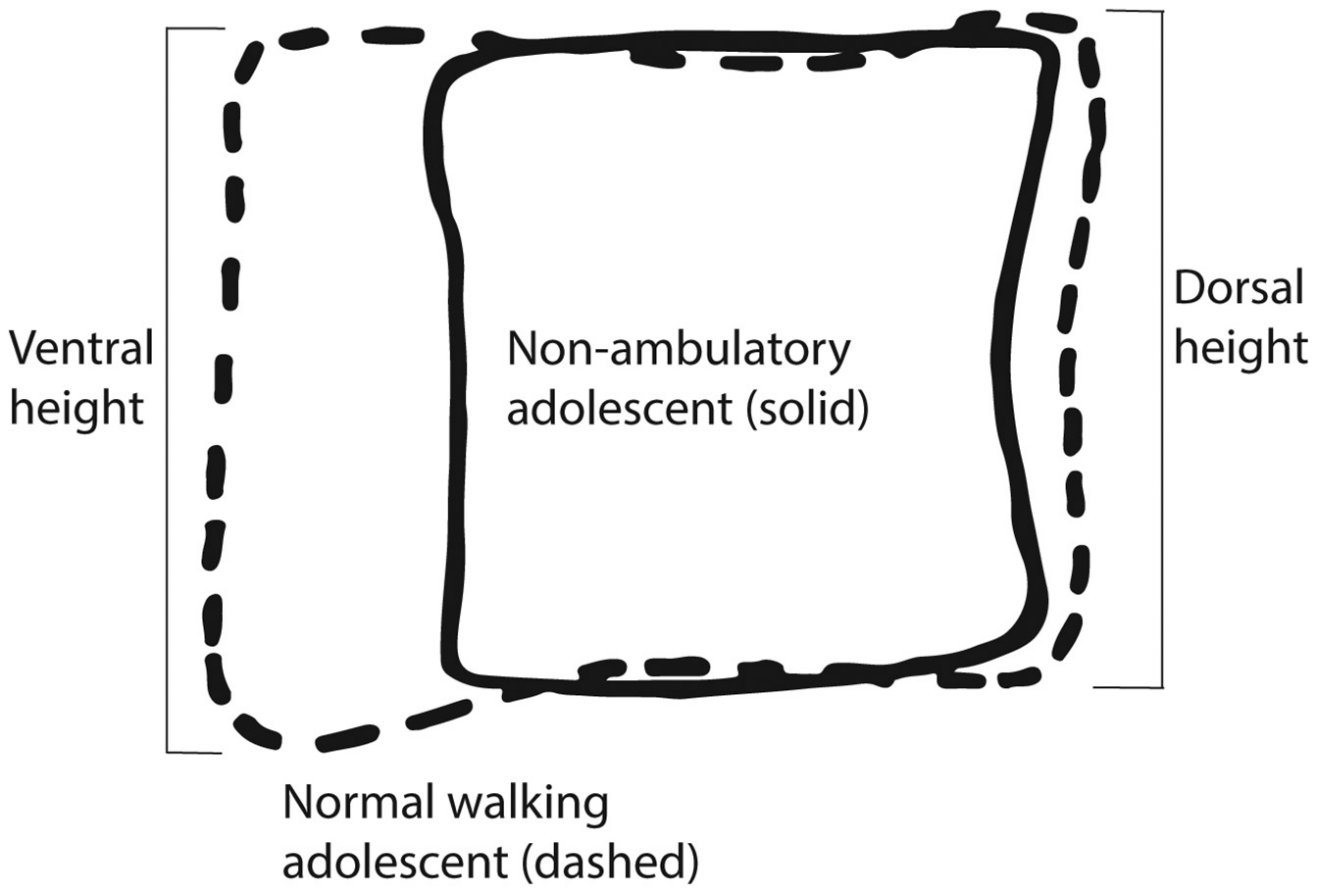
Comparison of lower lumbar vertebrae of nonambulatory and normal walking adolescent modern humans. The nonambulatory individual (solid tracing) attained a similar vertebral height as the normal walking individual (dashed tracing) but did not gain a similar degree of dorsoventral expansion through ontogeny as the normal walking individual. The lumbar vertebra of the nonambulatory individual also presents only a slight dorsal wedge, whereas the normal individual demonstrates significant dorsal (lordotic) wedging. The ontogeny of vertebral body shape, therefore, allows for the plasticity of vertebral wedging. Modified from ref. (37).

Much work has been done since the late 19th century on the development and plasticity of soft tissue and bony structures. Children are born with a relatively straight, slightly kyphotic spine, but some degree of lumbar lordosis is present at the lumbo-sacral border in fetuses irrespective of age (32), suggesting that its initial presence is genetic rather than plastic (i.e. mechanically induced). The largest increase in lordosis, though, occurs in the first 3 years of life during which time children develop the ability to sit up and walk (33). Lordosis angle increases until adolescence (13–15 years), with one study suggesting that it continues to increase until 20 years of age (34). This is consistent with the cessation of vertebral growth beginning around age 17–19 and completing by age 25 (35, 36). Vertebrae change in size and shape through ontogeny, with most craniocaudal growth complete by 10 years of age; however, the vertebral growth plates are present until around 25 years of age, allowing some growth and change in vertebral body shape until that time (35). Taylor (37) showed that nonambulatory adolescents did not develop normal lower lumbar vertebra shape. Whereas centrum cranio–caudal height was not different between nonambulatory and normal walking/weight-bearing adolescents, dorsoventral length was significantly and drastically shorter in the nonambulatory sample (Figure 5). In sum, while some aspect of centrum height appears to be genetically determined, the dorsal and especially ventral heights of the vertebral body are affected by mechanical action during development and into adulthood.

Along with other soft tissue structures, the intervertebral discs clearly play a role in anatomical spinal curvature. During development, the relative contributions of the vertebral bodies and the intervertebral discs to the overall form of the lordotic angle changes significantly. In young children (2–4 years of age), the vertebral bodies account for 53% of overall lordosis, with 47% accounted for by the intervertebral discs; by the onset of adulthood (17–20 years of age), the discs make up 80% of the overall lordosis angle and the vertebral bodies account for just 20% of lordosis (38). Despite the relatively low contribution of vertebral body shape to lumbar lordosis in adults, vertebral body indices and WAs contribute significantly to the lordosis angle (39, 40). Additionally, the evolution of the lumbar angle in humans seems to have resulted largely from modification in wedging of the vertebral bodies. Been et al. (40) showed that although intervertebral disc wedging contributes to spinal curvature in both macaques and humans, it accounts for just 17% of the difference between the two species, whereas vertebral body wedging accounts for the remaining 83% of the difference in overall lordosis angles between humans and macaques. In Japanese macaques trained as juveniles to walk bipedally, increased lordosis is achieved mainly via intervertebral disc wedging as opposed to vertebral body wedging (41). Therefore, the *capacity* to take on significant vertebral body wedging during development explains the larger lordosis angles that humans can achieve, whereas nonhominins may only be capable of increasing lordosis significantly via the intervertebral discs.

Activity during life, including posture, should therefore be expected to affect not just soft tissue morphology (e.g. wedging of the intervertebral discs) in humans, but also vertebral body shape, especially prior to and up through early adulthood. Reliance on furniture and especially sitting influences back posture (42) and might be expected to affect soft tissue structures and the development of bony morphologies. Conversely, or in tandem, use of squatting postures, which involves constant muscle contraction, in preindustrial populations (43), and lack thereof in furniture-reliant postindustrial societies, may also affect the development of vertebral morphologies. Similarly, recent sedentary humans demonstrate more gracile long bone internal structure than nonsedentary humans (44, 45). Gokhale (46), in a popular book about back pain, suggests that a correlation between the maintenance of cultural posture practices and healthy back posture exists and contrasts an exaggerated spinal curve (excessive kyphosis and lordosis) in industrial societies with healthier spines in more “traditional” societies where cultural practices lead to better posture in a variety of activities. If some degree of lumbar morphology is culturally and environmentally mediated, larger scale population differences would be expected irrespective of geographic ancestry. Any or all of these activity- and posture-related factors might account for the difference observed between preindustrial and postindustrial lifestyle samples.

We expect that individuals in our preindustrial lifestyle sample possessed similar degrees of *overall* lumbar lordosis as individuals in our postindustrial sample. However, we might expect differences in trunk musculature and intervertebral discs in individuals living contrasting lifestyles. Low back pain is reportedly much lower in low-income countries where traditional agriculture practices and other rural, high activity occupations are common (47). Other factors (e.g. lifespan longevity and cultural differences) represent confounding factors (reviewed in (47)); however, even within low-income countries, higher rates of low back pain are associated with urban areas and especially in “enclosed workshop” settings where employees maintain tedious and painful work postures (e.g. constant sitting on stools in a forward leaning position) (47). Castillo and Lieberman (48) suggest that the prevalence of back pain in contemporary humans is due to low levels of physical activity and consequent reduced spinal loading compared to preindustrial humans. Inadequate trunk muscle control, strength, and endurance are associated with injuries and low back pain, as are poor postures in both sitting and standing (reviewed in (49)). We suggest that underdeveloped soft tissue structures and prolonged sitting and other problematic postures can result in exaggerated bony lordosis. In contrast, the maintenance of healthy postural traditions and active lifestyles would produce adequate trunk musculature equipped to support lordotic posture without extra bony compensation. These hypotheses require careful testing outside the bounds of this study that considers activity and posture throughout development, but especially while vertebral body growth occurs (childhood through early adulthood).

### Implications for Inferring Lordosis in Neandertals

Recent authors have argued that Neandertals lack a discernable lumbar lordosis and instead possessed a “straight,” largely kyphotic lower back (5–8, 26). Two recent studies challenge this hypothesis, finding Neandertals to be more similar to modern humans. García-Martínez et al. (9) find ∑WA in Neandertals to fall well within the range of modern humans, but argue that the Neandertal *pattern* of vertebral wedging is distinct. Haeusler et al. (23) dismiss the Kebara 2 Neandertal as developmentally abnormal but find evidence for normal pelvic incidence (and therefore, inferred modern humanlike lumbar lordosis) in La Chapelle-aux-Saints 1 and other indications of lumbar lordosis in additional Neandertal individuals. Our results suggest that Neandertals indeed possess high ∑WA and low ∑AP, but not outside the range of modern humans and not significantly different from males who lived preindustrial lifestyles (Figures 2 and 3). In fact, at individual lumbar levels, Neandertals fall well within the 95% PIs of preindustrial males and demonstrate similar patterns of change in wedging across vertebrae (Figure 4). Preindustrial people were almost certainly characterized by lumbar lordosis, achieved in large part through soft tissue morphologies (core musculature, ligamentous attachments, and dorsally wedged intervertebral discs). Evidence of lordosis-related pathologies (e.g. Baatstrup disease) in Shaindar 3 (50) and La Chapelle-aux-Saints 1 (23) further suggest that Neandertals demonstrated lumbar lordosis. Finally, given that sexual dimorphism in lumbar wedging is present in modern humans and potentially also in *Australopithecus* (10, 11), female Neandertals would be expected to demonstrate more dorsal (lordotic) wedging than the inferred male individuals included in this study.

### Conclusions and Implications

Our results support previous work suggesting that modern human males and females present significantly different degrees of vertebral body wedging at most lumbar levels and in ∑WA and ∑AP, with an important caveat: context of the remains matters. Postindustrial samples were significantly different from preindustrial lifestyle samples in both ∑WA and ∑AP in sex-specific comparisons. Whereas preindustrial males showed the most ventral (kyphotic) wedging and postindustrial females showed the most dorsal (lordotic) wedging, preindustrial females and postindustrial males are intermediate and overlap significantly. We found no significant differences associated with geography (in the preindustrial samples) or ancestry (in the postindustrial samples), in contrast with previous studies. This is due in part to nonconsideration of lifestyle differences between samples in previous studies. For example, Cunningham's (14) European samples were all derived from postindustrial cadavers, whereas nonEuropeans were sampled from populations living preindustrial lifestyles. Given the results presented here, it is essential that fossil hominins and preindustrial modern humans are not compared to samples from sedentary, industrialized populations, but rather to the remains of individuals that engaged in more active, traditional lifestyles. Rather than invoking innate human population differences in a complex anatomical structure like lumbar lordosis, researchers should first attempt to explore hypotheses of plasticity in skeletal structures. Future studies could expand the samples included in this study, both in terms of sample size depth and comparative breadth, the latter by including humans living in climatic extremes, and additionally study spinal curvature in living people with different lifestyles from birth.

## Supplementary Material

pgab005_Supplemental_Files

## Data Availability

All analyzed data are included in the manuscript and/or supporting information.
