# Supplementary material for: Inferring lumbar lordosis in Neandertals and other hominins
Source: PNAS Nexus. 2022 Mar 2;1(1):pgab005. doi: 10.1093/pnasnexus/pgab005 (PMC9801964; doi:10.1093/pnasnexus/pgab005)
Supplement: pgab005_Supplemental_Files [file pgab005_supplemental_files.zip › PNASNEXUS-PNASNEXUS-2021-00121-s01.docx]

Supplementary Information

Inferring lumbar lordosis in Neandertals and other hominins

Scott A. Williams, Iris Zeng, Glen J. Paton, Christopher Yelverton, ChristiAna Dunham, Kelly R. Ostrofsky, Saul Shukman, Monica V. Avilez, Jennifer Eyre, Tisa Loewen, Thomas C. Prang, Marc R. Meyer


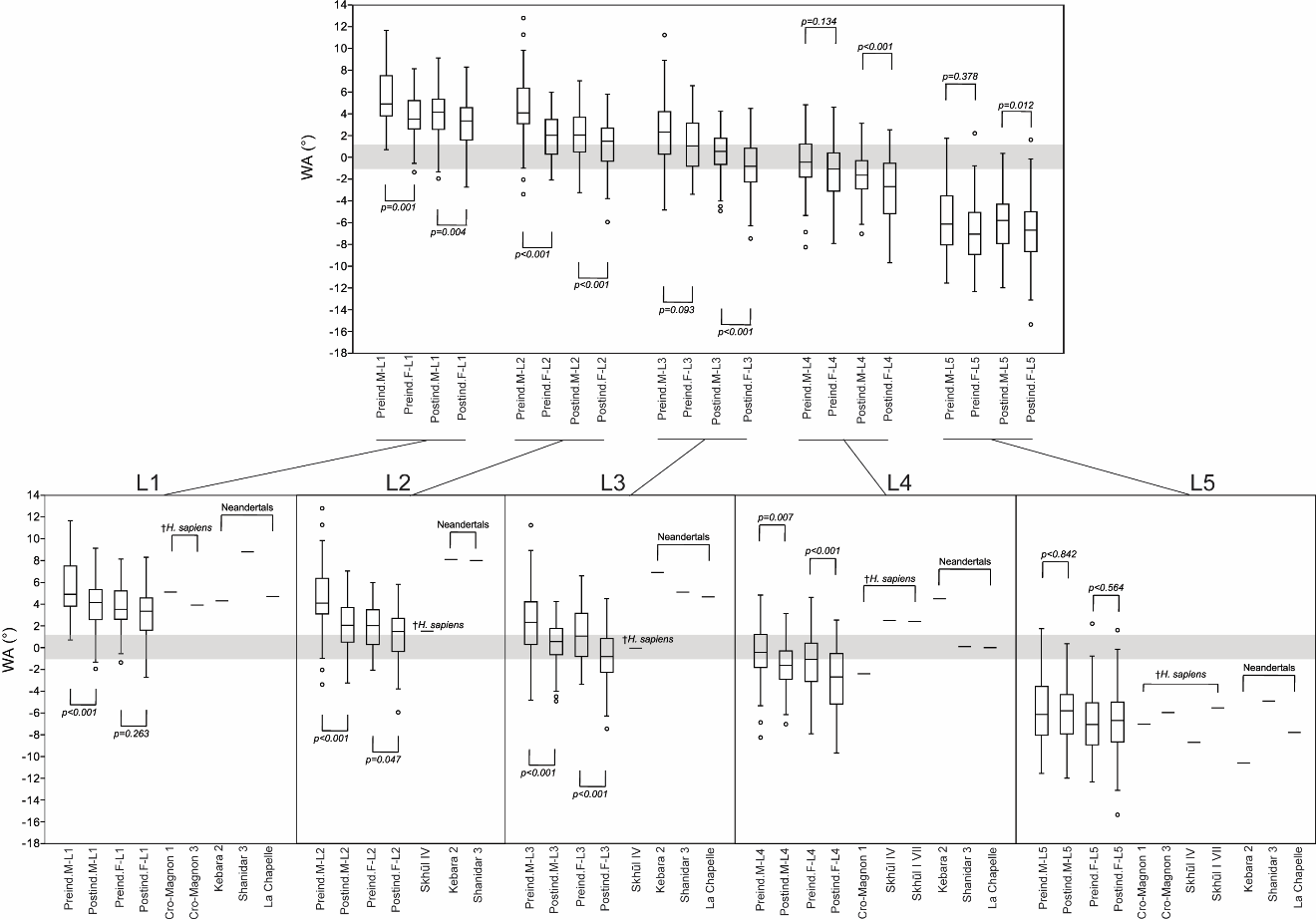


**Figure S1.** Lumbar vertebral body wedging angles (WA) in human groups and fossil hominins. Sex comparisons (M = males, F = female) in postindustrial (“Postind.”) and preindustrial (“Preind.”) samples across five lumbar vertebra levels. Comparisons at individual lumbar vertebra levels: first (L1) to last (L5) lumbar vertebrae WA, with fossils included when relevant. P-values of Tukey pairwise comparisons are shown. The shaded area highlights relatively neutral wedging (-1° to 1°).

**
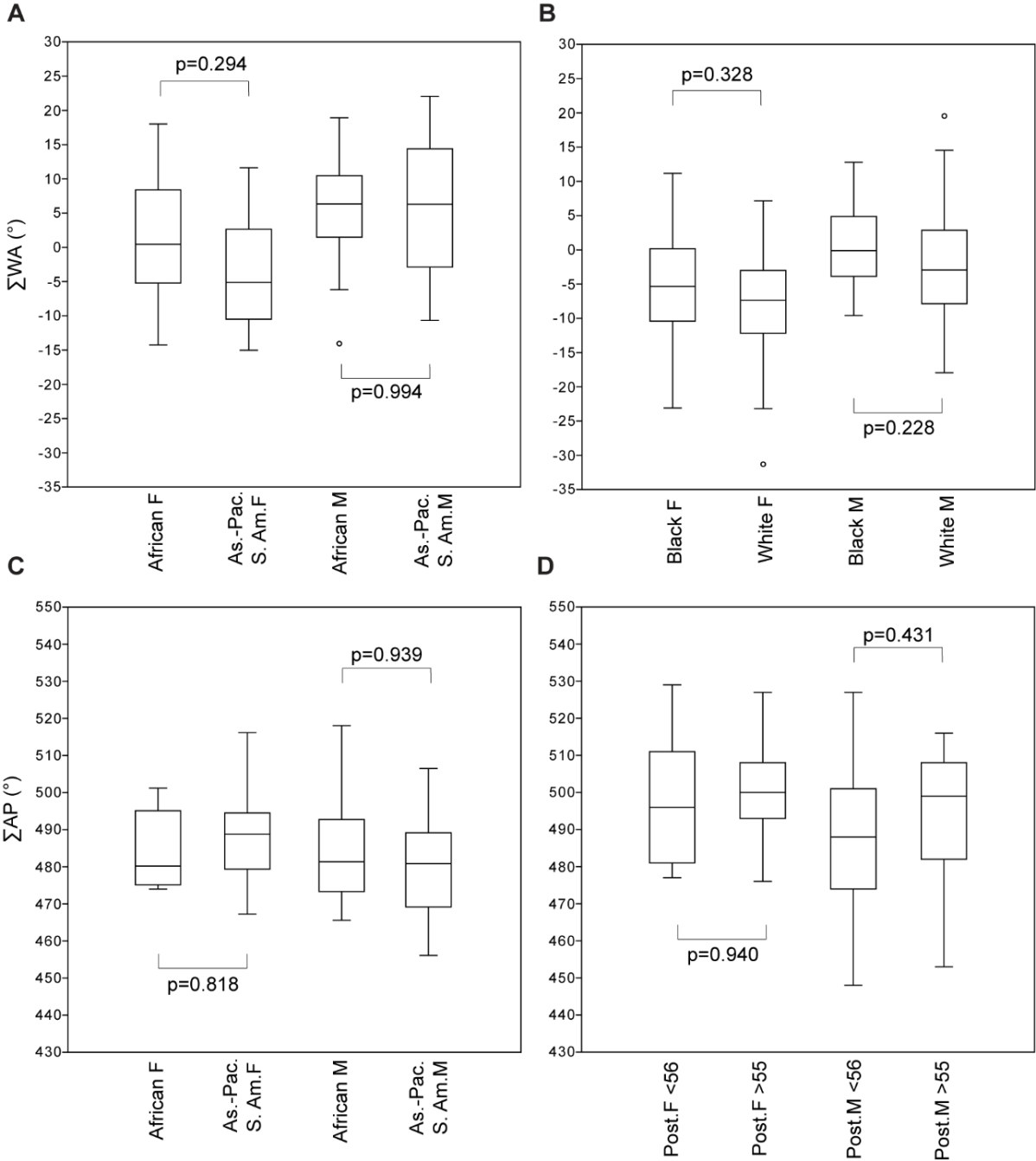
**

**Figure S2.** ∑WA and sum of lumbar vertebra inferior articular process angles (∑AP) in human groups. **(A)** ∑WA in sex-specific geographical ancestry groups in the preindustrial sample (As.-Pac./S. Am. = Asia Pacific/Suth American). **(B)** ∑WA in sex-specific ethnicity groups composing the postindustrial (“Post.”) sample (U.S. and South Africa). **(C)** ∑AP in sex-specific geographical ancestry groups in the preindustrial sample. **(D)** ∑AP in sex-specific U.S. postindustrial sample

from UTK and Texas State collections. P-values of Tukey pairwise comparisons are shown.

**
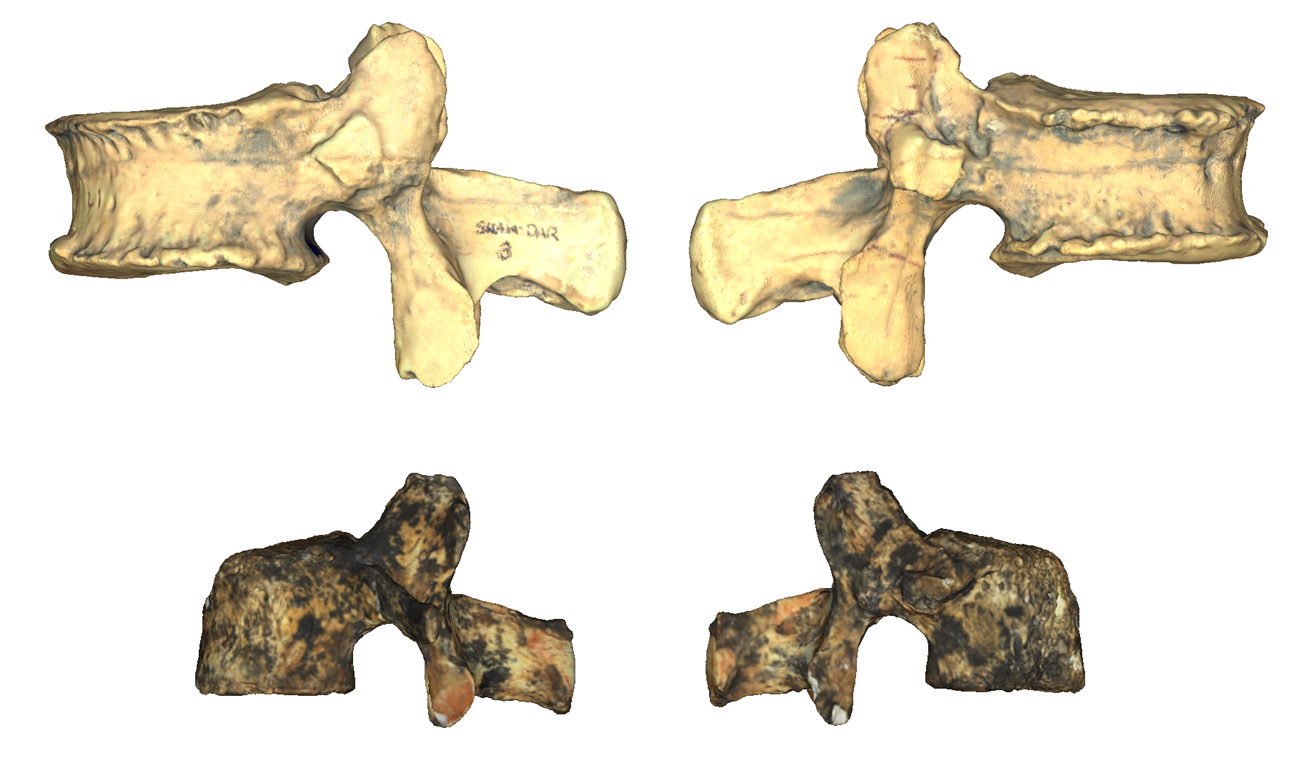
**

**Figure S3.** The last thoracic vertebrae of Shanidar 3 as defined using Schultz criteria (1). Note the bilateral presence of costal facets, located very dorsally in a position where lumbar transverse processes would attach on a lumbar vertebra. In this study, we use this vertebra as L1 since it is the fifth to last presacral vertebra.

**Table S1**. Descriptive statistics of first lumbar (L1) vertebral wedging angles.

| Group | N | Mean (std dev) | 95% PI lower | 95% PI upper |
| --- | --- | --- | --- | --- |
| Postindustrial | 254 |  |  |  |
| Females | 127 | 3.20 (2.21) | -1.13 | 7.53 |
| Males | 127 | 3.97 (2.07) | -0.10 | 8.04 |
| Preindustrial | 78 |  |  |  |
| Females | 31 | 3.70 (2.31) | -0.83 | 8.23 |
| Males | 47 | 5.63 (2.65) | 0.41 | 10.78 |
| † *H. sapiens* | 2 | 3.36 (2.20) | -0.96 | 7.67 |
| Neandertals | 3 | 5.93 (2.49) | 1.05 | 10.84 |
| † Fossil *H. sapiens*: This is a mixed-sex sample consisting of some individuals of unknown sex. | | | | |

**Table S2**. Descriptive statistics of second lumbar (L2) vertebral wedging angles.

| Group | N | Mean (std dev) | 95% PI lower | 95% PI upper |  |
| --- | --- | --- | --- | --- | --- |
| Postindustrial | 254 |  |  |  |  |
| Females | 127 | 1.23 (2.10) | -2.88 | 5.35 |  |
| Males | 127 | 2.15 (2.22) | -2.19 | 6.50 |  |
| Preindustrial | 78 |  |  |  |  |
| Females | 31 | 2.06 (2.08) | -2.02 | 6.14 |  |
| Males | 47 | 4.41 (3.31) | -2.08 | 10.90 |  |
| † *H. sapiens* | 3 | 0.51 (3.95) | -3.45 | 4.46 |  |
| Neandertals | 2 | 8.05 (0.71) | 6.66 | 9.44 |  |
| † Fossil *H. sapiens*: This is a mixed-sex sample consisting of some individuals of unknown sex. | | | | | |

**Table S3**. Descriptive statistics of third lumbar (L3) vertebral wedging angles.

| Group | N | Mean (std dev) | 95% PI lower | 95% PI upper |
| --- | --- | --- | --- | --- |
| Postindustrial | 254 |  |  |  |
| Females | 127 | -0.80 (2.26) | -5.23 | 3.56 |
| Males | 127 | 0.36 (1.86) | -3.29 | 4.01 |
| Preindustrial | 78 |  |  |  |
| Females | 31 | 1.26 (2.56) | -3.75 | 6.27 |
| Males | 47 | 2.38 (3.11) | -3.71 | 8.48 |
| † *H. sapiens* | 2 | 0.89 (1.25) | -1.57 | 3.34 |
| Neandertals | 3 | 5.55 (1.19) | 3.22 | 7.88 |
| † Fossil *H. sapiens*: This is a mixed-sex sample consisting of some individuals of unknown sex. | | | | |

**Table S4**. Descriptive statistics of fourth lumbar (L4) vertebral wedging angles.

| Group | N | Mean (std dev) | 95% PI lower | 95% PI upper |
| --- | --- | --- | --- | --- |
| Postindustrial | 254 |  |  |  |
| Females | 127 | -2.90 (2.75) | -8.28 | 2.48 |
| Males | 127 | -1.55 (2.01) | -5.48 | 2.39 |
| Preindustrial | 78 |  |  |  |
| Females | 31 | -1.49 (2.92) | -3.88 | 4.23 |
| Males | 47 | -0.52 (2.77) | -5.94 | 4.90 |
| † *H. sapiens* | 4 | 0.22 (2.58) | -4.84 | 5.27 |
| Neandertals | 3 | 1.53 (2.57) | -3.51 | 6.57 |
| † Fossil *H. sapiens*: This is a mixed-sex sample consisting of some individuals of unknown sex. | | | | |

**Table S5**. Descriptive statistics of fifth lumbar (L5) vertebral wedging angles.

| Group | N | Mean (std error) | 95% PI lower | 95% PI upper | |
| --- | --- | --- | --- | --- | --- |
| Postindustrial | 254 |  |  |  | |
| Females | 127 | -6.80 (2.86) | -12.40 | -1.20 | |
| Males | 127 | -5.95 (2.50) | -10.85 | -1.05 | |
| Preindustrial | 78 |  |  |  | |
| Females | 31 | -6.47 (3.12) | -12.59 | -0.35 | |
| Males | 47 | -5.86 (2.91) | -11.56 | -0.16 | |
| † *H. sapiens* | 4 | -6.85 (1.31) | -9.42 | -4.28 | |
| Neandertals | 3 | -7.76 (2.85) | -13.35 | -2.17 | |
| † Fossil *H. sapiens*: This is a mixed-sex sample consisting of some individuals of unknown sex. | | | | |  |

**References**

1. A. Schultz, *Vertebral column and thorax* (Karger Medical and Scientific Publishers, 1961).
